# Selenium-Enriched *E. coli* Bacteria Mitigate the Age-Associated Degeneration of Cholinergic Neurons in *C. elegans*

**DOI:** 10.3390/antiox13040492

**Published:** 2024-04-20

**Authors:** Palina Zytner, Anne Kutschbach, Weiye Gong, Verena Alexia Ohse, Laura Taudte, Anna Patricia Kipp, Lars-Oliver Klotz, Josephine Priebs, Holger Steinbrenner

**Affiliations:** 1 Institute of Nutritional Sciences, Nutrigenomics Section, Friedrich Schiller University Jena, D-07743 Jena, Germany; palina.buccellato@uni-jena.de (P.Z.); anne.kutschbach@uni-jena.de (A.K.); weiye.gong@uni-jena.de (W.G.); verena.ridolfi@uni-jena.de (V.A.O.); lars-oliver.klotz@uni-jena.de (L.-O.K.); 2 Institute of Nutritional Sciences, Department of Nutritional Physiology, Friedrich Schiller University Jena, D-07743 Jena, Germany; laura.taudte@uni-jena.de (L.T.); anna.kipp@uni-jena.de (A.P.K.)

**Keywords:** selenium metabolism, selenite, SEMO-1, stress, neurodegeneration, aging, antioxidant, prooxidant

## Abstract

Selenium (Se) is an essential trace element for humans and animals, but high-dose supplementation with Se compounds, most notably selenite, may exert cytotoxic and other adverse effects. On the other hand, bacteria, including *Escherichia coli* (*E. coli*), are capable of reducing selenite to red elemental Se that may serve as a safer Se source. Here, we examined how a diet of Se-enriched *E. coli* bacteria affected vital parameters and age-associated neurodegeneration in the model organism *Caenorhabditis elegans* (*C. elegans*). The growth of *E. coli* OP50 for 48 h in medium supplemented with 1 mM sodium selenite resulted in reddening of the bacterial culture, accompanied by Se accumulation in the bacteria. Compared to nematodes supplied with the standard *E. coli* OP50 diet, the worms fed on Se-enriched bacteria were smaller and slimmer, even though their food intake was not diminished. Nevertheless, given the choice, the nematodes preferred the standard diet. The fecundity of the worms was not affected by the Se-enriched bacteria, even though the production of progeny was somewhat delayed. The levels of the Se-binding protein SEMO-1, which serves as a Se buffer in *C. elegans*, were elevated in the group fed on Se-enriched bacteria. The occurrence of knots and ruptures within the axons of cholinergic neurons was lowered in aged nematodes provided with Se-enriched bacteria. In conclusion, *C. elegans* fed on Se-enriched *E. coli* showed less age-associated neurodegeneration, as compared to nematodes supplied with the standard diet.

## 1. Introduction

The essential trace element and micronutrient selenium (Se) is often designated as an antioxidant, which is, however, only true after its incorporation into antioxidant selenoenzymes [1,2]. In foodstuffs and dietary supplements, Se occurs in several chemical forms: organic Se compounds comprise selenocysteine, as well as selenomethionine and other methylated Se-containing amino acids, while the inorganic Se compounds constitute salts such as selenite and selenate [1,3]. Humans and animals may metabolize those Se compounds to hydrogen selenide (H_2_Se), the precursor for the biosynthesis of selenoproteins, many of which are enzymes that contain selenocysteine at their active site and participate in protection against oxidative stress. Most biological actions of Se are mediated through selenoproteins, and an increase in their biosynthesis and/or activity is thought to underlie the effects of dietary Se supplementation that are considered conducive to health in individuals with suboptimal Se status. In particular, Se and selenoproteins are essential for the function of the immune and the reproductive system, as well as the brain [1,2,3,4,5].

However, the “safe window” for dietary Se supply is narrow: The European Food Safety Authority (EFSA) recommends a daily intake of 70 µg Se for adults, whereas an intake of less than 30 µg Se/day is associated with Se deficiency. The upper tolerable intake level for adults has recently been set to 255 µg Se/day by the EFSA Panel on Nutrition, Novel Foods and Food Allergens [6]. Se intoxication may manifest in garlic-like malodor, hair loss and brittle nails. In animal studies, liver, cardiovascular, gastrointestinal and reproductive toxicities have been associated with Se oversupply [7]. Se compounds differ in their toxicity. In particular, selenite that is often included in dietary supplements may act as a prooxidant at high doses, exerting considerable cytotoxicity in cultured cells due to redox cycling [8,9].

Microbes, including yeast and various bacteria, are capable of converting selenite to less toxic forms of Se. In this regard, the microbial reduction of selenite to red elemental Se that may accumulate as intracellular deposits within a range of nano-to-low micrometer size has recently received attention. Biogenic Se nanoparticles are discussed as promising dietary supplements, due to their high bioavailability and biological activity, as well as their low toxicity [10,11,12]. Se nanoparticles produced by the gut bacterium and probiotic *Lactobacillus casei* (*L. casei*) have been reported to alleviate intestinal epithelial barrier dysfunction in vitro, as well as in vivo, employing cultured human colon cells and piglets, respectively, as model systems [13,14]. Moreover, supplementing the food with *L. casei* bacteria, which had been grown before in selenite-containing culture medium to induce the production of Se nanoparticles, counteracted cognitive dysfunction in a mouse model of Alzheimer’s disease (AD) [15]. The neuroprotective potential of both probiotics and Se has also been explored in a number of human studies with elderly individuals and AD patients, yielding mixed results, however [1,16,17]. Interestingly, co-supplementation with a mixture of probiotic bacteria and Se has been found to improve cognitive function in AD patients, as compared to the placebo group, as well as to the group who received only Se [18]. Even though the number of included participants was rather small in this pilot study, its observations may hold a promise that some gut microbiotas are capable of producing neuroprotective Se metabolites.

The use of the model organism *Caenorhabditis elegans* (*C. elegans*) in aging research has a long track record, yielding insights into genetic and environmental factors, including nutrition, that affect organismal aging, as well as enabling tests of anti-aging approaches [19,20]. Moreover, *C. elegans* is an excellent in vivo model for investigations of how the microbial conversion of dietary components such as Se may affect neuronal functions and neurodegeneration, as these nematodes rely on bacteria for feeding and their simple nervous system, comprising 302 neurons, is well-understood. Major molecular features such as neurotransmitters and their receptors, as well as the age-associated decline in neuronal functions, are evolutionarily conserved in *C. elegans* and in mammals [21]. Selenite has been reported to exert both beneficial and adverse effects on cholinergic neurons of the nematodes, depending on the applied dose [22,23]. Exposure of the worms to millimolar concentrations of selenite resulted in acute toxicity and developmental retardation. Compared to selenite, methylated Se-containing amino acids were only slightly toxic, even at the highest applied dose of 50 mM [24]. On the other hand, exposure to 100 µM of selenite, selenomethionine or Se-methylselenocysteine during hatching later protected the worms from oxidative stress induced by *tert*-butyl hydroperoxide. This beneficial effect was mediated by a Se-induced increase in the activity of the selenoenzyme TRXR-1, a thioredoxin reductase [25] and the only selenoprotein in *C. elegans* [26].

Here, we show that *Escherichia coli* (*E. coli*) OP50 bacteria, the common food source for *C. elegans* in the laboratory setting [27,28], accumulated red elemental Se if they were grown in culture medium supplemented with 1 mM selenite. In nematodes fed on the Se-enriched bacteria, indications of mild sublethal intoxication, such as growth retardation and delayed production of progeny, were observed. In response to the high Se supply, the expression of the selenium-binding protein orthologue with methanethiol oxidase activity (SEMO-1) [29,30], which is thought to serve as a Se buffer under conditions of environmental exposure to high Se concentrations [31], was elevated. The cholinergic neurons of aged nematodes fed on Se-enriched bacteria were less damaged, as compared to nematodes of the same age who had received the standard *E. coli* diet.

## 2. Materials and Methods

### 2.1. Culture and Treatment of E. coli OP50 Bacteria

The *E. coli* OP50 strain was obtained from the *Caenorhabditis* Genetics Center (CGC, University of Minnesota, Minneapolis, MN, USA), which is supported by the National Institutes of Health (NIH)–Office of Research Infrastructure Programs. The bacteria were routinely cultured in double yeast extract and tryptone (DYT) medium (Carl Roth; Karlsruhe, Germany). To obtain Se-enriched bacteria, the DYT medium was supplemented with 1 mM sodium selenite (Sigma-Aldrich; Munich, Germany), and the bacteria were allowed to grow for 48 h at 37 °C under constant agitation. Thereafter, the bacteria were pelleted by centrifugation; the bacterial pellet was washed twice with and resuspended in DYT medium. The bacteria destined for the standard *E. coli* diet of *C. elegans* were grown and treated in the same manner, but without the addition of selenite to the DYT medium.

### 2.2. Determination of the Se Content in Bacterial Growth Media and Pellets

Se-enriched culture media and bacteria were collected at the beginning and at the end of the 48-h growth period. After harvesting, the bacterial pellets were washed, weighed and lysed via sonication. The Se content in the samples was measured in duplicates by total reflection X-ray fluorescence (TXRF) spectrometry, using a bench-top TXRF spectrometer (S2 Picofox, Bruker Nano, Berlin, Germany) with 1 mg/L Yttrium (Merck/Millipore; Darmstadt, Germany) as internal standard, as described [32,33].

### 2.3. Maintenance and Treatment of C. elegans

The *C. elegans* strains Bristol N2 (wild-type) and LX929 (*vsIs48[unc-1::GFP]*; with its cholinergic neurons tagged by green fluorescent protein (GFP)) were obtained from the CGC. The *C. elegans* strain LOK158 (*pY37A1B.5::Y37A1B.5::GFP*, *+ pRF 4(rol-6(su1006)*), overexpressing GFP-tagged SEMO-1 (formerly known as Y37A1B.5) under the control of the *semo-1* promoter, was established as previously described [29]. Worms were routinely grown, maintained and treated at 20 °C on nematode growth medium (NGM) agar plates carrying a lawn of *E. coli* OP50 bacteria as food source, as described [27,29]. Experiments were initiated with worms in their L1 larval stage, starting 1 day after synchronization. Worms were randomly assigned to a group, which was fed either the standard *E. coli* OP50 diet or Se-enriched *E. coli* OP50, for the specified time. During long-term experiments, the worms were washed off the plates with S-buffer daily and were transferred to freshly prepared NGM agar plates spotted with the respective food source [29].

### 2.4. Determination of Body Length and Area of the Nematodes

Body length and area of wild-type (Bristol N2) worms were measured using a Ti Eclipse fluorescence microscope with NIS-Elements software, version AR 4.6 (Nikon, Düsseldorf, Germany). On the specified consecutive days, 10 randomly selected nematodes of each group were transferred into a drop of a sodium azide (10 mM) solution placed on a microscope slide and covered with coverslips. Photographs of single worms viewed with transmitted light were taken and analyzed, as previously described [29].

### 2.5. Fecundity of the Nematodes

Wild-type nematodes at the L1 stage were fed for 2 days either the standard or the Se-enriched *E. coli* OP50 diet. Thereafter, 10 single worms of each group were isolated on new agar plates carrying the respective bacterial lawn in order to lay eggs. This procedure was repeated daily for a total of 10 days. The remaining progeny was counted daily [29].

### 2.6. Determination of the Pharyngeal Pumping Rate of the Nematodes

Wild-type nematodes were fed for 4 days either the standard or the Se-enriched *E. coli* OP50 diet, starting at the L1 stage. Thereafter, 50 nematodes of each group were randomly selected and placed into a drop of *E. coli* OP50 suspension on a microscope slide coated with 3% agarose. For each nematode, a 10 s video of its pharyngeal movements was recorded, using a Ti Eclipse fluorescence microscope with NIS-Elements software (Nikon). Pharyngeal movements during these 10 s were counted, and the data were converted to the number of pharyngeal movements per minute.

### 2.7. Bacteria Choice Assay

The preference of the nematodes for the bacterial food offered to them was explored according to previously described behavioral assays [34,35]. An NGM agar plate of 90 mm in diameter was divided into four sections, and *E. coli* OP50 and Se-enriched *E. coli* OP50 bacteria were spotted on opposite sections with 3 cm distance from the center of the plate. Thereafter, a drop of S-buffer containing wild-type nematodes (at day 5 after the L1 stage) was placed in the exact epicenter. The number of transferred nematodes was counted at the beginning of the experiment. After a period of 3, 6 and 9 h, the nematodes in the initial spot, as well as the worms that had moved to any of the bacterial lawns, were counted again.

### 2.8. Fluorescence Microscopy of the GFP-Tagged C. elegans Strains LOK158 and LX929

Analysis of GFP fluorescence in the worms was performed as previously described [29,31], using a Ti Eclipse fluorescence microscope (Nikon) equipped with NIS-Elements software and appropriate filters (ex. 472 ± 30 nm; em. 520 ± 35 nm).

Nematodes of the LOK158 strain were fed for 3 days either the standard or the Se-enriched *E. coli* OP50 diet, starting at their L1 stage. Thereafter, 20 randomly selected nematodes of each group were transferred into a drop of a sodium azide (10 mM) solution placed on a microscope slide and covered with coverslips. For quantitation of the GFP signal intensity, worms were marked as regions of interest (ROI). Total GFP intensity and area of each ROI were then measured, background signal subtracted, and GFP intensity normalized to worm area to obtain the relative GFP fluorescence intensity for each worm [29,31].

Nematodes of the LX929 strain were fed for up to 12 days either the standard or the Se-enriched *E. coli* OP50 diet, starting at their L1 stage. On the specified consecutive days, 30 randomly selected nematodes of each group were transferred into a drop of a sodium azide (10 mM) solution placed on a microscope slide and covered with coverslips. Photographs of the GFP-tagged cholinergic neurons were taken in the area between the pharynx and vulva of the worms. The nerve cords were categorized as either healthy (no visible damage), mildly damaged (showing scattered minor knots) or severely damaged (showing multiple big knots or ruptures). For each day of observation, the proportion of normal (N), mildly damaged (MD) and severely damaged (SD) neurons was calculated. In addition, a neurodegeneration index (NDI) was calculated according to the following formula: NDI = (0 × N + 1 × MD + 2 × SD)/(N + MD + SD).

### 2.9. Statistical Analysis

Means were calculated from three independent experiments, and error bars represent the standard error of the mean (S.E.M.). For statistical analysis, GraphPad PRISM software, version 8.0.1 (GraphPad Software; San Diego, CA, USA), was used. Analysis of statistical significance was performed by Student’s *t*-test or one-way ANOVA with Dunnett’s multiple comparisons tests, as indicated, with *p* < 0.05 considered statistically significant.

## 3. Results and Discussion

### 3.1. Accumulation of Red Elemental Se in E. coli OP50 Bacteria Exposed to High Doses of Selenite

As for humans and animals, the trace element Se is both essential and toxic for *E. coli* bacteria, depending on the environmental Se concentration and the chemical form of Se [36]. *E. coli* has three selenocysteine-containing selenoenzymes, whose biosynthesis requires sufficient Se supply and is upregulated upon supplementation of the bacterial culture medium with selenite [37]. In addition, *E. coli* has been shown to metabolize selenite to volatile methylated Se compounds and to red elemental Se [36,37,38,39]. The reduction of selenite to elemental Se, which accumulates intracellularly as red deposits along the bacterial plasma membrane [40], serves as a mode of detoxification, allowing *E. coli* to grow in the presence of millimolar selenite concentrations [36,37]. For the *E. coli* K12 strain, reddening of the culture medium has been observed upon exposure to selenite concentrations ranging from 50 µM to 30 mM, with inhibition of more than 50% of bacterial growth at ≥5 mM selenite only [37].

**Figure 1:**
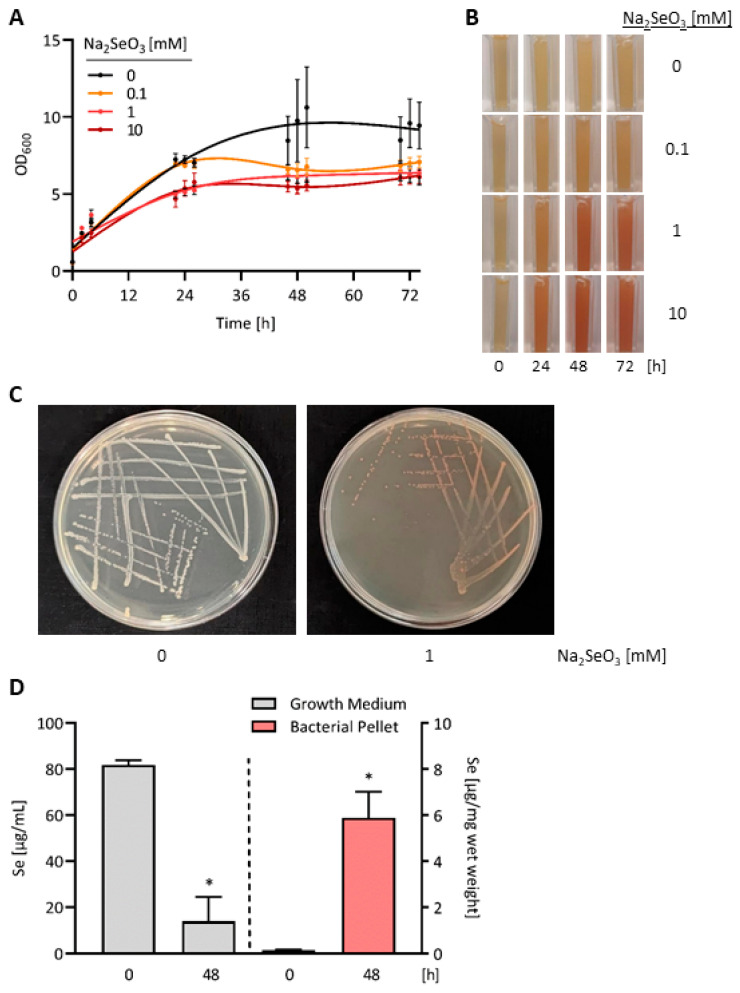
Accumulation of red elemental selenium in selenite-treated *E. coli* bacteria. (**A**) *E. coli* OP50 bacteria were grown in DYT medium supplemented with different concentrations of sodium selenite. Bacterial growth was assessed by measuring the optical density (OD) of the *E. coli* suspensions at 600 nm at the indicated time points. Data are shown as means ± S.E.M. from three independent experiments. (**B**) Representative photographs, showing aliquots of the selenite-treated *E. coli* suspensions in cuvettes. (**C**) Photographs of *E. coli* OP50 bacteria streaked on LB agar plates without added selenite (left plate) or supplemented with 1 mM selenite (right plate) and grown at 37 °C for 48 h. (**D**) *E. coli* OP50 bacteria were grown for 48 h in DYT medium supplemented with 1 mM sodium selenite. Se contents of the growth medium and the bacterial pellet were measured by total reflection X-ray fluorescence spectrometry (TXRF) at the beginning and at the end of the experiment. Data are depicted as means ± S.E.M. from three independent experiments; statistical analysis was performed using a paired *t*-test (* *p* < 0.05).

To define the conditions for production of Se-enriched bacteria as food source of *C. elegans*, *E. coli* OP50 bacteria were cultivated for up to 3 days in the presence of different selenite concentrations. After one day, bacterial growth was marginally affected at 100 µM selenite and inhibited by ~30% at 1 mM or 10 mM selenite in the culture medium. At none of the three tested selenite concentrations was the growth of *E. coli* OP50 inhibited by more than 50% after two or three days of treatment (Figure 1A). Exposure to 100 µM selenite induced a slight and barely detectable reddening of the bacterial culture. Both 1 mM and 10 mM selenite caused pronounced reddening of the bacterial culture, which appeared to be saturated after 2 days of treatment (Figure 1B). Moreover, cultivation of *E. coli* OP50 for 2 days on lysogeny broth (LB) agar plates containing 1 mM selenite also resulted in reddening of the bacteria (Figure 1C). The Se content of the bacterial pellet was increased 41.3-fold after cultivation of *E. coli* OP50 for 2 days in DYT medium containing 1 mM selenite, as the bacteria accumulated the bigger part of Se added to the culture medium (Figure 1D). Thus, Se-enriched bacteria for all further experiments were obtained by cultivating *E. coli* OP50 for 48 h with 1 mM selenite in their growth medium.

### 3.2. Influence of Se-Enriched E. coli OP50 Bacteria on Vital Parameters of C. elegans

As part of their diet, humans and animals may ingest various chemical forms of the micronutrient Se, which differ in regard to bioavailability, metabolization and toxicity [1,3,5]. Generally, high doses of inorganic Se species appear to be more toxic than organic Se species. This is also true for the model organism *C. elegans*, for which selenite has been found to be less bioavailable but more toxic as compared to the amino acids selenomethionine and Se-methylselenocysteine [24]. For acute (30 min) exposure of *C. elegans* to selenite in liquid culture, an LD_50_ of 13.1 mM was determined [24]. Chronic sublethal toxicity was observed, if worms were kept in axenic medium containing 200 µM selenite, resulting in delayed growth without impairment of reproduction [41]. Culturing worms on agar containing 20 µM selenite also resulted in growth retardation and decreased brood size; in contrast, worms held on agar supplemented with 10 nM or 50 nM selenite developed faster and showed elevated fertility [23].

The microbial conversion of selenite to red elemental Se has been reported to increase its bioavailability and to lower its toxicity. The resulting biogenic Se particles are currently discussed as promising novel Se compounds with possible application as dietary supplements, as well as in agriculture, medicine and cosmetics [42,43]. Elemental Se particles of nanometer to micrometer size, which were produced and isolated from selenite-treated *Staphylococcus carnosus* bacteria, have been found to decrease the viability of an agriculturally relevant nematode, *Steinernema feltiae* [44]. For our study, we took advantage of a special aspect of the physiology of the nematode *C. elegans*, who uses live bacteria as its main food source. Micronutrients such as Se can be metabolized and converted by the bacteria, prior to their delivery to the worms through digestion of the consumed bacteria [45]. Therefore, we did not extract the deposits of red elemental Se, which had accumulated in the selenite-treated *E. coli* OP50 bacteria. Instead, the Se-enriched bacteria were fed directly to the nematodes, and their influence on growth, fecundity, food intake and feeding habits of *C. elegans* was explored in comparison to the standard *E. coli* OP50 diet.

**Figure 2:**
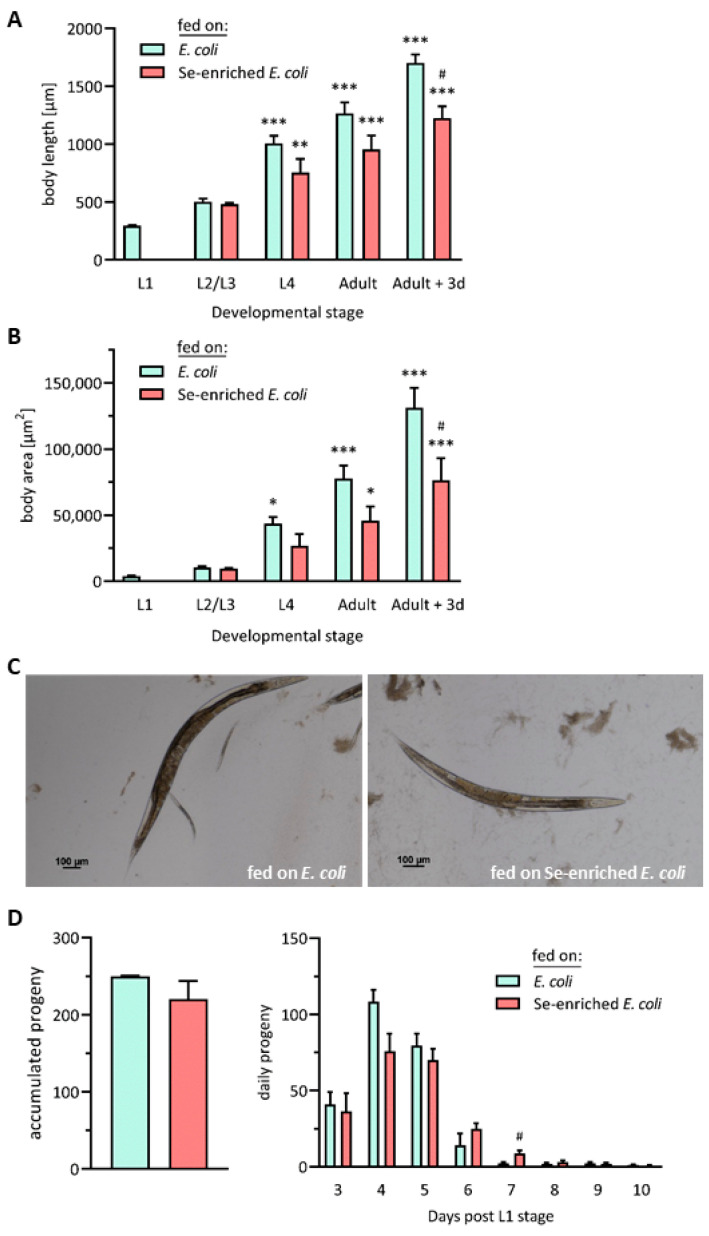
Developmental retardation of *C. elegans* fed a diet of Se-enriched *E. coli* bacteria. Bristol N2 (wild-type) worms at the L1 stage were supplied with either the standard *E. coli* OP50 diet or with Se-enriched *E. coli* OP50 for up to three days after reaching adulthood. Body length (**A**) and body area (**B**) of 10 worms from each of the specified consecutive developmental stages were determined by microscopy. Data are depicted as means ± S.E.M. from three independent experiments; statistical analysis was performed using one-way ANOVA (* *p* < 0.05, ** *p* < 0.01, *** *p* < 0.001 vs. L1 worms; # *p* < 0.05 vs. worms fed on the standard *E. coli* diet). (**C**) Representative photographs of nematodes which received the standard *E. coli* OP50 diet (left panel) or Se-enriched *E. coli* OP50 (right panel) from the L1 stage until 3 days of adulthood. (**D**) Larvae produced by *C. elegans* fed either the standard *E. coli* OP50 diet or the Se-enriched bacteria. The total (left panel) and daily (right panel) numbers of offspring were measured for up to 10 days after the worms reached the L1 stage. Data are depicted as means ± S.E.M. from four independent experiments; statistical analysis was performed using an unpaired *t*-test with Welch’s correction (# *p* < 0.05 vs. worms fed on the standard *E. coli* diet).

Starting at L1 stage, the growth of nematodes of the Bristol N2 (wild-type) *C. elegans* strain was monitored until day 3 after reaching adulthood (day 6 post-L1 stage) by consecutive measurements of their body length and area. As expected, both the body length and area of the nematodes strongly increased over the sequence of their developmental stages. However, these increases were more pronounced in the group receiving the standard *E. coli* OP50 diet, as compared to the worms fed on Se-enriched bacteria. Growth retardation of nematodes fed on the Se-enriched bacteria was observed for the first time at the L4 stage, even though the difference in body length and area between the two groups did not reach statistical significance before day 3 of adulthood (Figure 2A,B). From their outward appearance, adult worms who had received the Se-enriched bacteria looked generally smaller and slimmer (Figure 2C). Chronic sublethal Se toxicity has been reported to induce changes in the transcriptome of *C. elegans*, pointing to responses to oxidative and endoplasmic reticulum (ER) stress, as well as to impaired production of collagen and cuticle components [46]. This may explain the growth retardation observed here, as well as in studies where nematodes were exposed to high sublethal doses of selenite [23,41]. Moreover, there are several reports of lowered growth rates and body weight gain after feeding mammalian model organisms high doses of Se compounds [7]. Molecular mechanisms that are proposed to underlie Se toxicity comprise oxidative stress due to an increased production of reactive oxygen species (ROS) and glutathione depletion, suppression of methylation reactions due to depletion of S-adenosylmethionine (SAM) and reaction of Se with thiol moieties in proteins [6].

The fecundity of the nematodes was not impaired by feeding Se-enriched bacteria, as compared to the nematodes fed the standard *E. coli* diet; both groups produced a similar total number of offspring. Nevertheless, a trend to slower and prolonged production of larvae was observed in the group of worms fed on the Se-enriched bacteria (Figure 2D).

**Figure 3:**
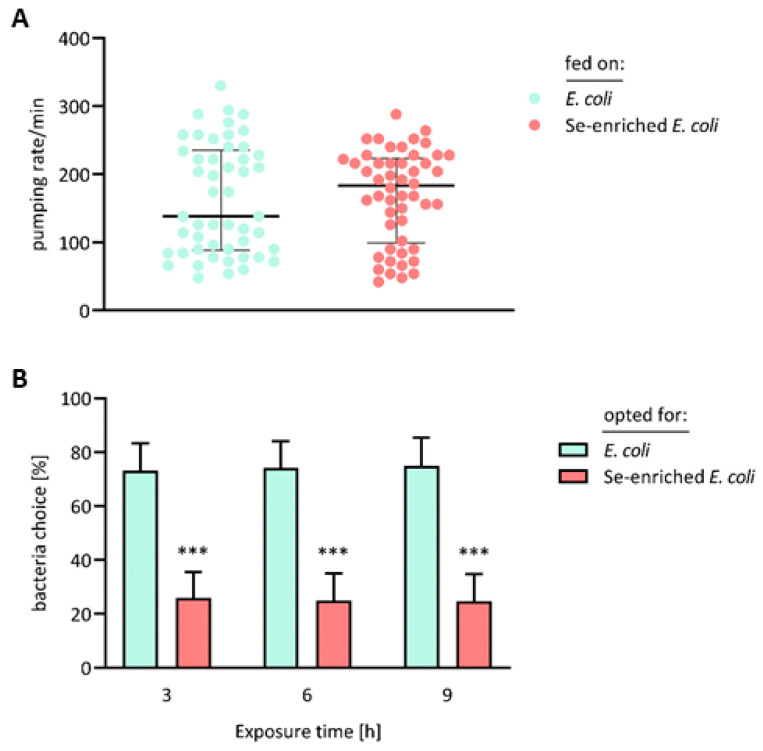
Food intake and preference of *C. elegans* fed on Se-enriched bacteria, as compared to the standard *E. coli* diet. (**A**) Bristol N2 worms, starting at L1 stage, received either the standard or the Se-enriched *E. coli* OP50 diet for 4 days. Thereafter, the pumping rate of 50 nematodes from each group was determined by recording videos of their pharyngeal movements under a microscope. Data are depicted as medians with interquartile ranges; there was no significant difference between the two groups, as shown by statistical analysis using an unpaired *t*-test with Welch’s correction. (**B**) Bristol N2 worms at the L1 stage received the standard *E. coli* OP50 diet for 4 days. Thereafter, they were placed in the epicenter of an agar plate, with lawns of *E. coli* OP50 and Se-enriched *E. coli* OP50 bacteria at opposite sections. For calculation of the relative bacteria choice by the nematodes, the total number of the transferred worms, as well as the number of worms which had opted for one of the food sources, was counted at the beginning of the experiment and after 3, 6 and 9 h, respectively. Data are depicted as means ± S.E.M. from four independent experiments; statistical analysis was performed using an unpaired *t*-test with Welch’s correction (*** *p* < 0.001 vs. worms fed on the standard *E. coli* diet).

*C. elegans* ingests bacteria by means of the pharynx, an organ specialized in filtering, concentrating and grinding food particles from their environment [28]. To explore whether the observed developmental retardation of worms which received the Se-enriched bacteria was due to limited food intake, the pharyngeal pumping movements of the nematodes were thus surveyed. However, there was no statistically significant difference between the pharyngeal pumping rates of worms to which the standard *E. coli* OP50 diet or the Se-enriched *E. coli* OP50 bacteria were offered (Figure 3A).

Even though the nematodes accepted the Se-enriched bacteria as a food source, the vast majority preferred their standard *E. coli* diet, if they had the choice: in the case that both food sources were available in equidistance, ~75% of the worms chose to graze on the lawn of *E. coli* not supplemented with selenite, while only ~25% of the worms chose the Se-enriched bacteria (Figure 3B). In its natural environment, *C. elegans* is capable of discriminating between pathogenic bacteria and bacterial food, whose intake supports faster growth, through olfactory recognition of bacterially released aversive and attractive volatile compounds [47,48,49]. Selenite-supplemented *E. coli* bacteria have been reported to release two volatile Se compounds, dimethyl selenide and dimethyl diselenide, as well as a volatile Se–sulfur compound, dimethyl thioselenide [39]. Presumably, these malodorous compounds are perceived by *C. elegans* as being repugnant.

### 3.3. Feeding Se-Enriched E. coli OP50 Bacteria Results in Elevated Levels of the Se-Binding Protein SEMO-1 in C. elegans

To date, two Se-containing proteins have been identified in *C. elegans*: (i) thioredoxin reductase TRXR-1, a selenoenzyme with selenocysteine in its active center [26], is required for removal of the old cuticle during molting of the worms [50]; and (ii) SEMO-1, an enzyme that oxidizes alkyl thiols such as methanethiol, binds Se most likely through one or multiple cysteine residue(s) in an analogous manner to its human orthologue selenium-binding protein 1 (SELENBP1) [29,30,32].

**Figure 4:**
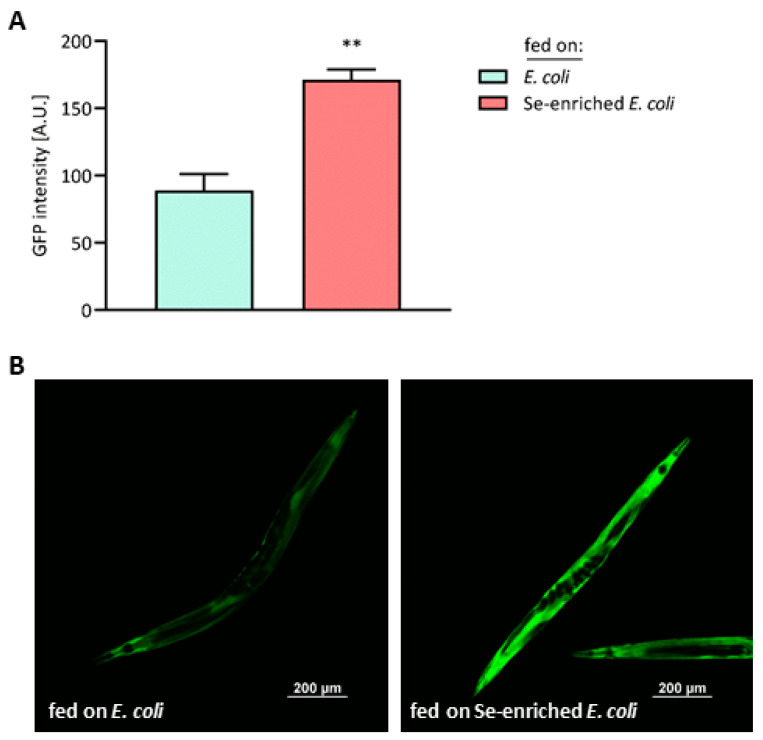
Elevated levels of the Se-binding protein SEMO-1 in *C. elegans* fed on Se-enriched *E. coli* bacteria. The transgenic *C. elegans* strain LOK158, overexpressing GFP-tagged SEMO-1 under control of the *semo-1* promoter, was supplied with either the standard *E. coli* OP50 diet or with Se-enriched *E. coli* OP50 for three days, starting at L1 stage. (**A**) GFP intensity, given as arbitrary units (A.U.), was determined by fluorescence microscopy, analyzing 20 worms from each group. Data are depicted as means ± S.E.M. from three independent experiments; statistical analysis was performed using an unpaired *t*-test with Welch’s correction (** *p* < 0.01). (**B**) Representative photographs of nematodes that received the standard *E. coli* OP50 diet (left panel) or Se-enriched *E. coli* OP50 (right panel).

Using the *C. elegans* reporter strain LOK158, i.e., worms overexpressing GFP-tagged SEMO-1 under control of the *semo-1* promoter, it was previously shown that SEMO-1 levels increase in response to exposure to high doses (≥100 µM) of selenite. Thus, SEMO-1 may serve as a Se buffer in worms, under conditions of intense environmental exposure due to high Se concentrations in the soil or in their food [31]. Consistent with this hypothesis, and using the same LOK158 reporter strain, SEMO-1 levels were strongly elevated in nematodes fed on Se-enriched *E. coli* bacteria (Figure 4A,B), suggesting that the red elemental Se accumulated in those bacteria was bioavailable to the nematodes. This is, however, a surrogate marker, which should be further substantiated by future analyses of the Se content and species in nematodes fed on the Se-enriched bacteria.

### 3.4. Se-Enriched E. coli OP50 Bacteria Mitigate the Progressive Damage of Cholinergic Neurons in Aging Nematodes

In humans, aging is accompanied by progressive functional decline of cholinergic neurons, characterized by dendritic, axonal and synaptic degeneration rather than neuronal cell death [51]. Similarly, cholinergic neurons of aging nematodes develop morphological defects, without cell loss [52,53]. Here, we monitored age-dependent neurodegenerative changes in *C. elegans* and the influence of Se-enriched *E. coli* bacteria thereon, making use of the LX929 transgenic strain that expresses a GFP reporter in all cholinergic neurons. This model has been applied before to investigate the neurotoxic actions of environmental chemicals [54,55].

**Figure 5:**
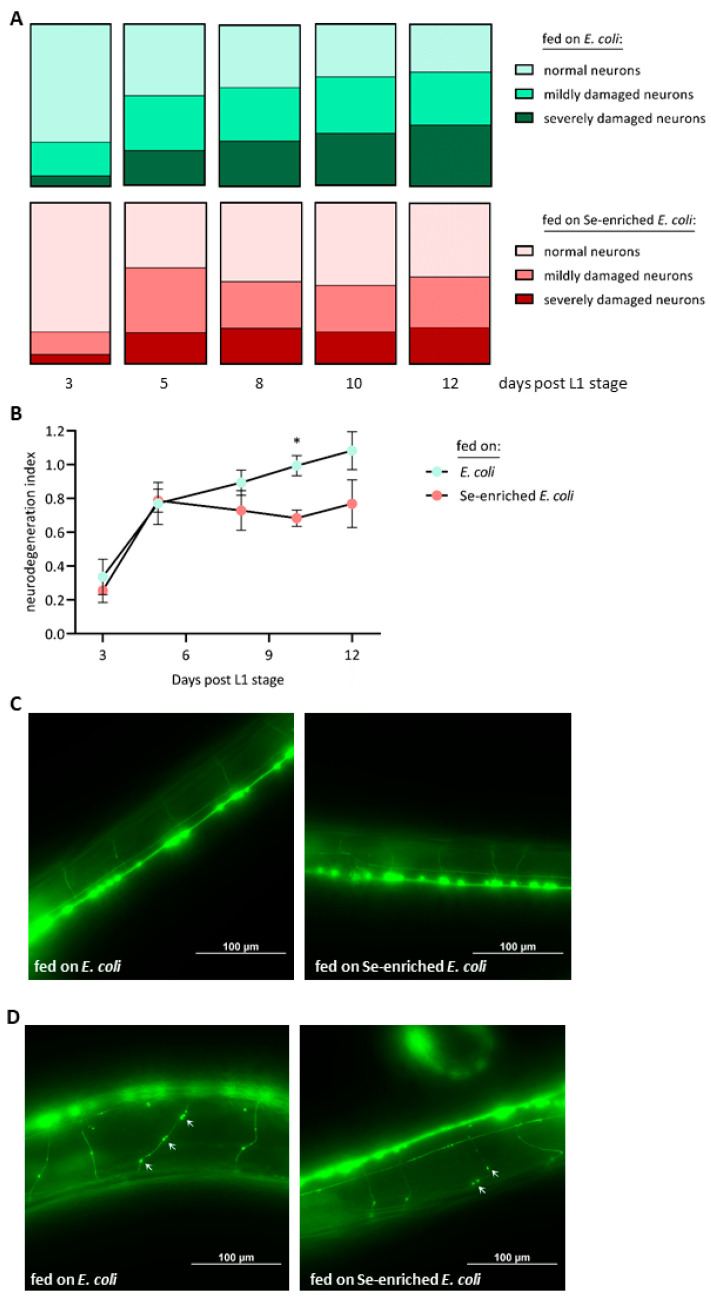
Less age-associated defects in the cholinergic neurons of *C. elegans* fed on Se-enriched *E. coli* bacteria. The transgenic *C. elegans* strain LX929, with its cholinergic neurons tagged by GFP, was supplied with either the standard *E. coli* OP50 diet or with Se-enriched *E. coli* OP50 for up to 12 days, starting at the L1 stage. The nerve cords of the nematodes were visualized by fluorescence microscopy. (**A**) At each of the specified days, the fractions of healthy, mildly damaged and severely damaged neurons of 30 nematodes from each group were calculated. Data are depicted as means from three independent experiments. (**B**) Neurodegeneration index, calculated by weighting the occurrence of mild and severe damages in the neurons. Data are depicted as means ± S.E.M. from three independent experiments; statistical analysis was performed using an unpaired *t*-test with Welch’s correction (* *p* < 0.05). (**C**) Representative photographs of young nematodes who had received the standard *E. coli* OP50 diet (left panel) or Se-enriched *E. coli* OP50 (right panel) for 3 days (post L1 stage). (**D**) Representative photographs of aged nematodes who had received the standard *E. coli* OP50 diet (left panel) or Se-enriched *E. coli* OP50 (right panel) for 12 days (post L1 stage). Note the lower extent of damage, e.g., lower numbers of knots (as marked by the white arrows), in the right panel.

The cholinergic neurons of the nematodes were visualized by fluorescence microscopy at consecutive points in time, from day 3 to day 12 after L1 stage. In this period, the fraction of normal neurons without any visible damage dropped from 73% to 29% in the worms fed the standard *E. coli* diet, while the fraction of neurons with severe defects (multiple knots and ruptures) continuously rose from 6% to 38% (Figure 5A). In comparison, delayed and lower occurrence of morphological defects of the cholinergic neurons and better preservation of undamaged neurons were observed in the group of worms fed on Se-enriched bacteria. In these worms, the fraction of severely damaged neurons rose from 6% to 23% only, and the fraction of normal neurons dropped from 80% to 46% within the period of record (Figure 5A). This neuroprotective effect of the Se-enriched bacteria is reflected in the calculated neurodegeneration index, which was lower for the group of worms fed on Se-enriched bacteria from day 8 post-L1 on, even though the difference between the two groups reached statistical significance only at day 10, due to high standard deviations (Figure 5B). Generally, the cholinergic neurons of young nematodes at day 3 post-L1 showed hardly any morphological defect, independent of their diet (Figure 5C). On the other hand, aged nematodes held on Se-enriched bacteria for 12 days since their L1 stage showed less signs of degeneration, as compared to aged nematodes supplied with the standard *E. coli* diet (Figure 5D).

Chronic exposure to Se, in the form of selenite, has previously been found to both support and repress cholinergic signaling in *C. elegans*, depending on the applied selenite concentrations [22,23]. Interestingly, sublethal toxic effects of selenite on development, growth and larvae production were coupled with adverse effects on the nervous system of *C. elegans* [23]. In contrast, we found that the Se-enriched *E. coli* bacteria were neuroprotective despite eliciting mild developmental retardation. This might suggest a hormetic effect with mild sublethal stress in young worms and subsequent induction of protective actions. A potential novel selenoprotein-independent mechanism of Se-mediated neuroprotection has only recently been pointed out by the observation that a key intracellular Se metabolite, hydrogen selenide, may donate electrons to ubiquinone to form ubiquinol, an intermediate in the mitochondrial respiratory chain [56]. This may implicate a Se-mediated support of mitochondrial ATP production [57], which is otherwise impaired in the aged brain and in the brain of patients suffering from neurodegenerative diseases such as AD [58]. Compared to *C. elegans* with only one selenoprotein [26], humans have 25 selenoproteins, including several antioxidant selenoenzymes [2,17]; thus, selenoproteins are likely to be more important for neuroprotective actions of Se in humans than in *C. elegans*. Interestingly, a low Se status of elderly individuals has been reported to be associated with faster cognitive decline and poor performance in tests assessing coordination and motor speed [59]. In humans, adequate dietary Se supply may thus contribute to counteract mild forms of the age-associated decline of brain functions, while no evidence for a hypothesized protective role of dietary Se supplements against AD has been provided to date [1,17,60].

## 4. Conclusions and Future Perspectives

**Figure 6:**
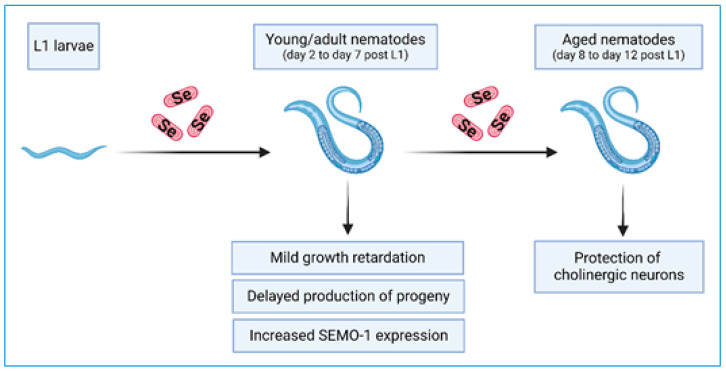
Effects of a diet of Se-enriched *E. coli* OP50 bacteria on young/adult and aged *C. elegans* observed in this study. Scheme created with Biorender.com (accessed on 15 April 2024).

Dietary Se supplementation is a “double-edged sword”, with beneficial effects for the health of Se-deficient humans and animals but an inherent risk for adverse effects and intoxication if Se is overdosed [1,2,3,4,5,6,7]. The risk/benefit relation of Se depends on the applied Se dose, as well as on the applied Se compound. In this regard, biogenic and non-biogenic particles of red elemental Se have recently attracted attention as less toxic and highly bioavailable Se compounds [10,11,12,13,14,15,42,43,44]. We show here that feeding the model organism *C. elegans* with Se-enriched *E. coli* bacteria that had accumulated red elemental Se provoked mild sublethal toxicity characterized by slight developmental retardation of the young animals. On the other hand, the diet of Se-enriched bacteria was neuroprotective in aged animals, lowering age-associated morphological defects in their cholinergic neurons (Figure 6). This dual outcome may suggest a hormetic effect.

In this pilot study, we provided evidence for the feasibility of our novel approach to feed nematodes with bacteria that had metabolized selenite and accumulated red elemental Se deposits. The use of a transgenic *C. elegans* strain with GFP-tagged cholinergic neurons enabled us to monitor the consecutive occurrence of morphological defects in the cholinergic neurons of aging worms and the effect of the Se-enriched bacterial diet on it. In future studies, functional parameters (e.g., pumping rate, motility, acetylcholine and/or acetylcholine esterase levels) should be analyzed as well in the aged worms. Moreover, our approach can be adopted to other *C. elegans* models available for research on neurodegeneration and -protection, such as transgenic strains that express GFP specifically in dopaminergic or in GABAergic neurons, as well as transgenic strains with expression of human β-amyloid peptide in neurons or muscles [61].

The molecular mechanism behind the neuroprotective effect of Se-enriched bacteria observed in the aged worms is another issue for future analyses. Several cellular key functions are dys- or downregulated in the aged brain and in the brain of persons suffering from neurodegenerative diseases. In fact, aging is considered the primary risk factor for the development of neurodegenerative diseases [62]. Signaling through the transcription factor nuclear factor erythroid 2-related factor 2 (Nrf2) that allows cells adaptive responses to environmental challenges such as ROS or xenobiotics becomes progressively impaired during aging, while its activation may mitigate features of AD, Parkinson’s disease (PD) and multiple sclerosis (MS) [63]. Interestingly, biogenic Se nanoparticles have recently been reported to activate Nrf2 and to increase the expression of several Nrf2 target genes [64], making the *C. elegans* orthologue SKN-1 a promising candidate for a molecular target of the neuroprotection through Se-enriched bacteria.

## Data Availability

The original contributions presented in the study are included in the article, further inquiries can be directed to the corresponding author/s.
